# Processability of a Hot Work Tool Steel Powder Mixture in Laser-Based Powder Bed Fusion

**DOI:** 10.3390/ma15072658

**Published:** 2022-04-04

**Authors:** Nick Hantke, Felix Großwendt, Anna Strauch, Rainer Fechte-Heinen, Arne Röttger, Werner Theisen, Sebastian Weber, Jan Torsten Sehrt

**Affiliations:** 1 Chair of Hybrid Additive Manufacturing, Ruhr-University Bochum, 44801 Bochum, Germany; jan.sehrt@ruhr-uni-bochum.de; 2 Chair of Materials Technology, Ruhr-University Bochum, 44801 Bochum, Germany; felix.grosswendt@ruhr-uni-bochum.de (F.G.); theisen@wtech.rub.de (W.T.); weber@wtech.rub.de (S.W.); 3 Leibniz Institute for Materials Engineering—IWT, 28359 Bremen, Germany; strauch@iwt-bremen.de (A.S.); fechte@iwt-bremen.de (R.F.-H.); 4 MAPEX Center for Materials and Processes, University of Bremen, 28359 Bremen, Germany; 5 Chair of New Manufacturing Technologies and Materials, University of Wuppertal, 42651 Solingen, Germany; roettger@uni-wuppertal.de

**Keywords:** additive manufacturing, powder mixing, alloying strategies for powder bed fusion of metals using a laser beam system, tool steel, laser reflectance

## Abstract

Powder bed fusion of metals using a laser beam system (PBF-LB/M) of highly complex and filigree parts made of tool steels is becoming more important for many industrial applications and scientific investigations. To achieve high density and sufficient chemical homogeneity, pre-alloyed gas-atomized spherical powder feedstock is used. For high-performance materials such as tool steels, the number of commercially available starting powders is limited due to the susceptibility to crack formation in carbon-bearing steels. Furthermore, scientific alloy development in combination with gas-atomization is a cost-intensive process which requires high experimental effort. To overcome these drawbacks, this investigation describes the adaption of a hot work tool steel for crack-free PBF-LB/M-fabrication without any preheating as well as an alternative alloying strategy which implies the individual admixing of low-cost aspherical elemental powders and ferroalloy particles with gas-atomized pure iron powder. It is shown that the PBF-LB/M-fabrication of this powder mixture is technically feasible, even though the partly irregular-shaped powder particles reduce the flowability and the laser reflectance compared to a gas-atomized reference powder. Moreover, some high-melting alloying ingredients of the admixed powder remain unmolten within the microstructure. To analyze the laser energy input in detail, the second part of the investigation focuses on the characterization of the individual laser light reflectance of the admixed alloy, the gas-atomized reference powder and the individual alloying elements and ferroalloys.

## 1. Introduction

PBF-LB/M has recently gained an increased scientific and economic importance and is, in many cases, considered to revolutionize current product design and development [1]. In the field of tool and mold making, additive manufacturing (AM) allows the production of complexly shaped tools and molds with minimal use of materials, and therefore a high level of resource efficiency. In addition, these tools can be temperature-controlled optimally and locally through internal cooling or heating channels which are located below the functional surface [2,3]. This temperature control allows faster cycle times, which increases both the efficiency of the respective value chain and the surface quality of the products [4]. Tools for the processing of metals and plastics are made of tool steels. In the case of hot forming or press hardening, hot work tool steels such as X38CrMoV5-1 (AISI H11) and X40CrMoV5-1 (AISI H13) are commonly used because of their high hardness, toughness, and wear resistance [5]. The material properties are determined by the microstructure in the heat-treated state. By hardening and tempering in the regime of secondary hardness, a microstructure consisting of tempered martensite with finely dispersed secondary carbides is formed [6].

The processing of these carbon martensitic tool steels using welding or additive manufacturing is challenging due to the high tendency of forming cold cracks [7]. Cold cracks form when the material cools locally from the process heat below the martensite start (M_S_) temperature. The formed martensite possesses high strength, thus residual stresses can only be reduced by local plastic deformation of the softer retained austenite (RA). If the residual stresses locally exceed the strength of the RA, cold cracking occurs. To counteract the cold crack formation of carbon martensitic steels during PBF-LB/M measures such as preheating of the build platform or multiple exposures are used [7,8,9]. Counteracting cold cracking by build platform preheating brings disadvantages such as pronounced oxygen-uptake of the powder bed. This limits the reuse of the powder for subsequent AM-processes [7,10]. Because of the challenging processability of carbon martensitic hot work tool steels, tools and molds are preferably produced from precipitation hardening steels, such as X5CrNiCuNb17-4-4 (17-4 PH) or X3NiCoMoTi18-9-5 (MS1) by PBF-LB/M [9,11]. However, these materials do not always provide the required hardness, toughness, and wear resistance to guarantee sufficient durability of the hot forming tool and are cost-intensive due to the high contents of alloying elements.

For this reason, there is a high need for the development of carbon martensitic steels possessing good processability by AM without additional procedural measures and which, for reasons of cost, have a low content of alloying elements. Such new carbon martensitic tool steels appropriate for AM processes can be derived using the concept of low transition temperature (LLT) steels. The LTT concept utilizes a targeted reduced M_S_ compared to conventional tool steels such as H13 to avoid cold cracking during rapid cooling conditions in welding or AM processes.

The martensite formation alone does not lead to cold cracks since the fcc → bcc transformation causes material expansion, which is associated with the reduction in previously formed residual tensile stresses during cooling. Moyer et al. investigated the volume expansion of this γ-α′-transformation by dilatometric measurements and found an increase in the volume of the material by 2.0% (0.19 mass% C) to 3.1% (1.01 mass% C) [12]. With an increase in the volume content of martensite, the expansion capacity of the entire matrix decreases and only the softer RA undergoes a local plastic deformation, which is accompanied by cold crack formation if the strength of the RA is exceeded. It can be concluded that the formation of martensite can be used to reduce the previously formed tensile stresses. Therefore, the M_S_ temperature must be adapted in such a manner that the maximum effect of this transformation plasticity is close to room temperature (RT). The reduction in the M_S_ temperature can be achieved by alloying with the elements Cr, Mn, and Ni [13]. Considering the work of Murata et al., Wang et al., and Eckerlid et al., residual tensile stresses formed during cooling from the process heat can even be converted into residual compressive stresses by this transformation plasticity effect [14,15,16].

Now, to support a cost-efficient method for the development of further LTT tool steels appropriate for AM and especially PBF-LB/M the use of admixed elemental and ferroalloy particles as feed-stock material is investigated in this work. Thereby, low-cost mechanically crushed particles possessing an aspherical shape are admixed with spherical gas atomized iron powder.

As Attar et al. and Spierings et al. stated, density and the mechanical properties of PBF-LB/M-fabricated parts are influenced by the homogeneity and the density of the applied powder layers and thus by the shape and particle size distribution of the used powders [17,18]. Spherical particles increase the packing density of powder particles and improve the flow properties of the powder [19,20]. While a certain amount of small powder particles improves the component properties, powders with a high proportion of small particles can have a negative effect on flowability, as these particles tend to form irregularly shaped agglomerates [18,19,21].

In general, a powder bed absorbs laser radiation better than a single powder particle or a smooth surface [22,23]. Between the powder particles of a powder bed, the incident laser beam is reflected multiple times, which increases the absorption [22,23,24,25]. With the use of ray tracing calculations, Boley et al. showed that the laser light absorption of powders depends on the particle size distribution [22]. While large particles reflect more radiation to the environment, small particles increase the absorption of the laser light [23,25].

The characteristics of the powder bed consisting of the considered powder mixture are expected to be changed compared to a commonly used spherical pre-alloyed powder feedstock. Until now, little research has been performed on the use of powder mixtures as feedstocks for laser-based AM, especially for PBF-LB/M. Schwendner et al. investigated the processing of elemental powder blends in a directed energy deposition process using a laser beam [26]. Roberts et al. mixed spherical Al powder with aspherical Si powder and processed this mixture via PBF-LB/M [27]. After the PBF-LB/M-process, Si particles were not completely dissolved in the Al matrix, which made a subsequent heat treatment necessary. Nevertheless, the work of Roberts et al. shows that the processing of powder mixtures in PBF-LB/M is possible [27].

In previous work, the nearly crack-free PBF-LB/M-fabrication of the considered hot work tool steel could already be shown, but the PBF-LB/M-fabrication of tool steels from powder mixtures has not been sufficiently investigated until now [28]. This work more deeply investigates the possibility of the PBF-LB/M-fabrication of a hot work tool steel powder mixture developed with the LTT alloy approach in mind. Powder properties and powder bed homogeneity of this powder blend as well as the resulting microstructures of the samples manufactured by PBF-LB/M are compared to those of gas-atomized pre-alloyed powder with the same alloy composition. In this investigation, the following scientific questions are addressed:

1. Is admixing of gas-atomized spherical pure iron powder with aspherical elemental powders and ferroalloys suitable for the PBF-LB/M fabrication of crack-free samples with high density?
2. Does utilization of the admixed alloy significantly interfere with the homogeneous powder particle distribution within the powder bed and the chemical homogeneity of the as-built samples?
3. To what extent does the admixing of aspherical particles with spherical iron powder influence the laser light reflection capacity of the powder bed? How much does powder particle size reduction decrease the reflectance of the laser light?

## 2. Materials and Methods

### 2.1. Alloying Strategy

In this work, a suitable carbon martensitic tool steel alloy, hereinafter referred to as ***H*** for hot work steel, was chosen for its low tendency for cold crack formation. The nominal chemical composition and the actual composition of the PBF-LB/M materials produced are given in Table 1.

The mechanical and tribological properties should be comparable to the common X40CrMoV5-1 hot work steel. The calculated M_S_ temperature according to Andrew’s approach [29,30] (Equation (1)) for the considered LTT hot work tool steel is about 193 °C, which is found to be suitable to counteract the formation of high residual tensile stresses during PBF-LB/M fabrication.

The considered steel was subsequently processed into dense samples using PBF-LB/M. Pre-alloyed powder was used for PBF-LB/M-fabrication and the gas-atomization (melting under argon and atomization with nitrogen) was performed at the Leibniz Institute for Materials Technology. Samples manufactured from this pre-alloyed powder using PBF-LB/M are referred to in the following as H-PRE (PRE = pre-alloyed). In addition, samples were generated from the powder mixture, which are referred to as H-MIX (MIX = mixture). Pure iron was molten in an argon-atmosphere and gas-atomized using nitrogen to a spherical powder. The iron powder was sieve-classified into a fraction of 20–63 µm and subsequently admixed with mechanically broken and sieve-classified ferroalloy and elemental particles (size 20–63 µm, see Table 2) to achieve the nominal chemical composition. Subsequently, the powder mixture was homogenized in a Turbula mixer (WAB group, Muttenz, Switzerland) for two hours. The chemical composition of the PBF-LB/M-processed powders was measured by optical emission spark spectrometry (type QSG750, OBLF GmbH, Witten, Germany) and the average mean value of five measurements was calculated.

(1) $$M_{S}=539-423\;\cdot \;\%C-30.4\;\cdot \;\%Mn-12.1\;\cdot \;\%Cr-17.7\;\cdot \;\%Ni-7.5\;\cdot \;\%Mo\;\;\;\;$$

### 2.2. Thermodynamic Calculations

Thermodynamic calculations for the hot work steel considered here were carried out using the ThermoCalc (TC) software (ThermoCalc AB, Stockholm, Sweden) in version 2017a. Calculations were performed with the database TCFE7 and the phases LIQUID (liquid), M6C (W-rich M_6_C), FCC_A1 (γ -Fe), FCC_A1#2 (V- and Ti-rich MC), M7C3 (Cr-rich M_7_C_3_), M23C6 (Cr-rich M_23_C_6_), M2C (Mo-rich M_2_C), and BCC_A2 (α-Fe). The SSOL4 database was used to calculate the solidus and melting temperatures of the ferroalloys used with less than 50 mass% of Fe and for pure elements (all phases were allowed). All calculations were carried out at an atmospheric pressure of 1000 mbar and a substance quantity of 1 mol. To illustrate the solidification sequence of the hot work tool steel during PBF-LB/M, solidification simulations were performed according to the approach by Scheil and Gulliver with the thermodynamic database TCFE7. These calculations were also carried out at a pressure of p = 1000 mbar and a quantity of 1 mol, beginning from a starting temperature of 2000 °C. For each iteration step, the temperature was reduced by 1 K.

### 2.3. Powder Characterization

The particle size distribution in the range from 0.02 to 2000 µm was determined in accordance with ISO 13320 (fineness characteristic diameter d) on the particle size measuring device Mastersizer Hydro 2000 G (Malvern Instruments Ltd., Malvern, UK). The device operated at a pressure of 2 bar and a frequency of 75 Hz using water as the dispersion medium. For determining the density distribution function, q_3_, a representative amount of powder (300 g) was investigated. Four powder samples from different locations of the powder container were taken. The determined density distribution function is related to the total amount of tested powder. In addition, the sphericity of the respective ferro-particles and pure elements was approximated by quantitative image analysis by calculating the circularity according to Riley [31]. The projection area of the powder particles was imaged at a magnification of 3500× by scanning electron microscopy (SEM) using MIRA3 (Tescan, Brno, Czech Republic). Bagheri et al. reported a yielded error of about 15.9% by approximating the sphericity by *ϕ*_Riley_ circularity [31].

An FT4 powder rheometer (Freeman Technology Ltd., Tewkesbury, UK) was used for further powder characterization. Five values of the H-PRE and H-MIX powders were compared: the conditioned bulk density (CBD), the basic flowability energy (BFE), the specific energy (SE), the flow rate index (FRI), and the pressure drop (PD). Prior to each measurement, a conditioning cycle was performed to remove precompaction and excess air from the powder samples and to establish a reproducible initial state. To perform a conditioning cycle, a rotating precision blade was moved into and out of a vessel containing the powder sample. The movement was carried out with a blade tip speed of 40 mm/s and a helix angle of 5°. The CBD was calculated from the ratio of the sample mass to the sample volume after a conditioning cycle has been performed. To measure the BFE and the SE, after the first conditioning cycle, a test cycle was run in which the rotating blade was moved into the powder sample at a blade tip speed of 100 mm/s and a helix angle of 5° and out of the powder sample with a blade tip speed of 40 mm/s and a helix angle of −5°. Then a new conditioning cycle was performed. This procedure was repeated seven times. The BFE represents the total energy needed to move the rotating precision blade into a powder-filled vessel during the seventh test cycle. The total energy is composed of the energy required for the rotary motion and the energy required for the axial movement into the powder sample. The SE was calculated from the ratio of the mean value of the energy required for the blade to move out of the powder-filled vessel during test cycles six and seven and the mass of the powder sample. The test for the FRI was carried out directly after the test for the BFE and the SE. After the last test cycle for the BFE and the SE, a new conditioning cycle was performed. A test cycle with a blade tip speed of 100 mm/s followed. Three further test cycles followed, in which the blade tip speed was reduced step by step using a step size of 30 mm/s. After each test cycle, a conditioning cycle was performed. The helix angle for all blade tip speeds was kept at 5° for the downwards movement and at −5° for the upwards movement. The FRI was calculated from the ratio of the energy needed to move the blade through the sample at blade tip speeds of 10 mm/s and 100 mm/s. To measure the pressure drop through a powder sample, the powder sample was compressed under normal stress with a vented piston after three conditioning cycles were performed. While the powder was compressed, air passed through the sample from the bottom of the vessel at a constant flow rate of 2 mm/s. The powder was gradually compressed at the normal stress of 1 kPa, 2 kPa, 4 kPa, 6 kPa, 8 kPa, 10 kPa, 12 kPa, and 15 kPa and the air pressure drop through the powder sample was recorded.

### 2.4. Laser Reflection Measurement

A Nicolet iS20 FTIR spectrometer (Thermo Fischer Scientific, Waltham, MA, USA) equipped with a white light source, a XT-KBr beam splitter, a MCT/A detector and a DiffusIR (Pike Technologies, Madison, WI, USA) was used to analyze the reflectance of the powder samples at RT. Thirty-two scans with a spectral resolution of 4 cm^−1^ were performed at MCT/A detector wavenumbers ranging from 4000 to 10,000 cm^−1^ in order to record the spectra. The wavenumber is the reciprocal of the wavelength. Therefore, the constant wavelength of 1070 nm of the utilized laser inside of the PBF-LB/M-system is represented by the wavenumber of 9345.8 cm^−1^. To ensure repeatable measurements three spectra were recorded for each sample. To gain information about the reflectance of different particle size fractions and the ingredients of the H-MIX powder each of the ingredients of the H-MIX powder was sieved into the three fractions <28 µm, 28–45 µm, and 45–63 µm. The reflectance of the feedstocks H-PRE, H-MIX, and each of the fractions were analyzed.

### 2.5. PBF-LB/M-Fabrication

For manufacturing of samples, the PBF-LB/M-system TruPrint 1000 (Trumpf GmbH + Co. KG, Ditzingen, Germany) equipped with a 200 W fiber laser with a wavelength of 1070 nm and a spot diameter of 30 µm was used. A rubber x-profile was used as recoating device. The PBF-LB/M-process took place under a nitrogen atmosphere, which offers the possibility to re-densify the fabricated samples by hot isostatic pressing (HIP) due to the nitrogen solubility of steels, which is not given for argon gas [32]. The oxygen content of the atmosphere was <0.1%.

Cube-shaped samples with an edge length of 4 mm were built to find suitable process parameters for the processing of the two powders. An inverted pyramid shape with a height of 1 mm and a base area of 3 × 3 mm^2^ was used to connect the cubic-shaped samples to the build platform. For both powders, twelve samples with varying scanning speeds and laser powers at a hatch distance of 60 µm and a layer thickness of 30 µm were manufactured. The scanning vectors followed a meandering shape, which was rotated by 90° after each layer. The scanning speed was varied between 720 and 990 mm/s in 90 mm/s increments and the nominal laser power was varied between 140 and 160 W in 10 W increments.

### 2.6. Microscopy

The cross-sections of the PBF-LB/M-samples were ground stepwise with SiC grinding paper from 320 to 1000 mesh and polished with diamond suspensions with a grain size from 3 to 1 μm. To determine the relative density of the samples, image analysis using a VHS 6000 digital microscope (Keyence Corporation, Osaka, Japan) was performed. Each of the investigated samples was prepared in three different layers. The density of each sample was determined from the mean value of the densities of these three layers. To investigate the particle morphology of the powders and the microstructures of the PBF-LB/M-densified samples a field-emission scanning electron microscopy of type MIRA3 (Tescan, Brno, Czech Republic) was used. The SEM operated at an acceleration voltage UA = 20 kV and a working distance WD = 9 mm. Micrographs were taken in secondary electron (SE) contrast if not stated otherwise. To determine inhomogeneities in the local chemical composition, energy dispersive X-ray spectrometry (EDS) was performed using an OXFORD X-Max 50 and the software Aztech in version AZtec 5.0 HF1 (Oxford instruments, Abingdon, UK). The working distance for EDS was set to 15 mm.

### 2.7. Determination of the Residual Stresses and the Retained Austenite Content

For the determination of the residual stresses as well as the RA contents of the PBF-LB/M-produced samples the X-ray diffractometer µx-360 (Pulstec, Hamamatsu, Japan) with a Cr X-ray tube was used. The samples were ground and polished (1 µm). During the measurement, the full Debye ring profiles from a single incident X-ray angle were detected and these profiles were software-converted into an intensity/2θ diagram. With the knowledge of the intensities of the martensite and austenite phases, the RA content could be quantified according to the Rietveld method. The residual stresses were determined three times in the x and y directions by the cos-α method, and the arithmetic mean was calculated from these results. A detailed description of this measurement method can be found in [33].

## 3. Results and Discussion

### 3.1. Powder Morphology and Powder Properties

**Figure 1:**
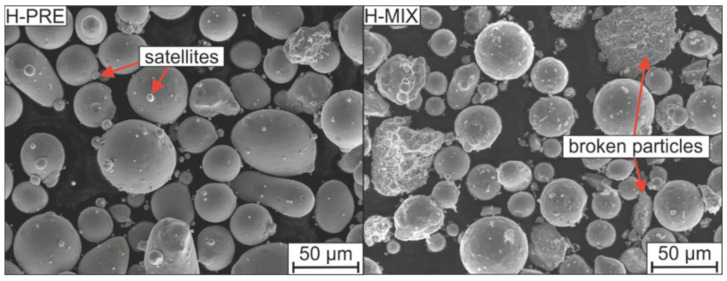
H-PRE and H-MIX powder morphologies.

The morphologies of the H-PRE and H-MIX powders are shown in Figure 1. Most H-PRE powder particles are spherical and show only a small number of satellites. However, some particles deviate from the spherical shape and even show irregular shapes, which could lead to decreased flowability (circularity S = 0.79). The gas-atomized Fe particles included in H-MIX show fewer satellites than the pre-alloyed particles of the H-PRE powder and are highly spherical with a circularity S = 0.85. These Fe particles are surrounded in the H-MIX powder by sharp-edged broken ferroalloy and elemental particles. The circularity of the broken particles ranges from S = 0.51 to S = 0.61 (Table 2) and does not depend on the respective particle size. Because of the high percentage of highly spherical Fe the overall circularity of the powder mix was calculated as S = 0.78. The circularity of the mixed powder measured by image analysis is S = 0.75 and correlates to the calculated mean value. Therefore, the circularity of H-MIX is only slightly inferior to the circularity of the gas-atomized pre-alloyed powder H-PRE. This promotes a slightly increased flowability of H-PRE.

**Figure 2:**
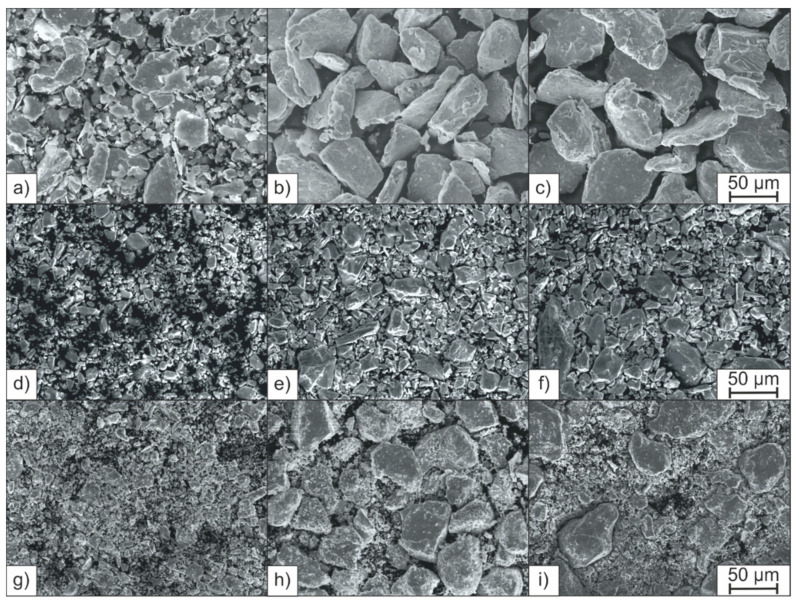
SEM images of the sieved fractions: (**a**) FeCrC < 28 µm, (**b**) FeCrC 28–45 µm, (**c**) FeCrC 45–63 µm, (**d**) Mn < 28 µm, (**e**) Mn 28–45 µm, (**f**) Mn 45–63 µm, (**g**) FeW < 28 µm, (**h**) FeW 28–45 µm, and (**i**) FeW 45–63 µm.

Adhering to the powder particles in H-MIX was a fine portion consisting of splinters of the broken raw materials. These fine particles were not separated from the powder mixture by sieving, which may be due to the tendency of fine particles to agglomerate [33]. The broken particles were sieved into three fractions (<28 µm, 28–45 µm, and 45–63 µm) and examined. Figure 2a–c shows the three fractions of FeCrC. After sieving, no smaller particles adhere to the particles of larger fractions, and between all three fractions a clear difference in particle size is visible in the SEM images. The SEM images of the fractions 28–45 µm and 45–63 µm of Mn (Figure 2d–f) do not show significant differences. Pure Cr shows a behavior similar to Mn. FeW and FeTi include very fine particles in the larger fractions after sieving which can be seen on the example of FeW in Figure 2g–i. As the example of FeW shows, finer particles can remain in the larger fractions of some materials. The fractions sieved this way, therefore, do not correspond to the specified particle sizes. For clarity, only the images of the FeCrC, Mn, and FeW particles are shown as examples in Figure 2 to describe the different distributions of particles sizes observed after sieving.

**Figure 3:**
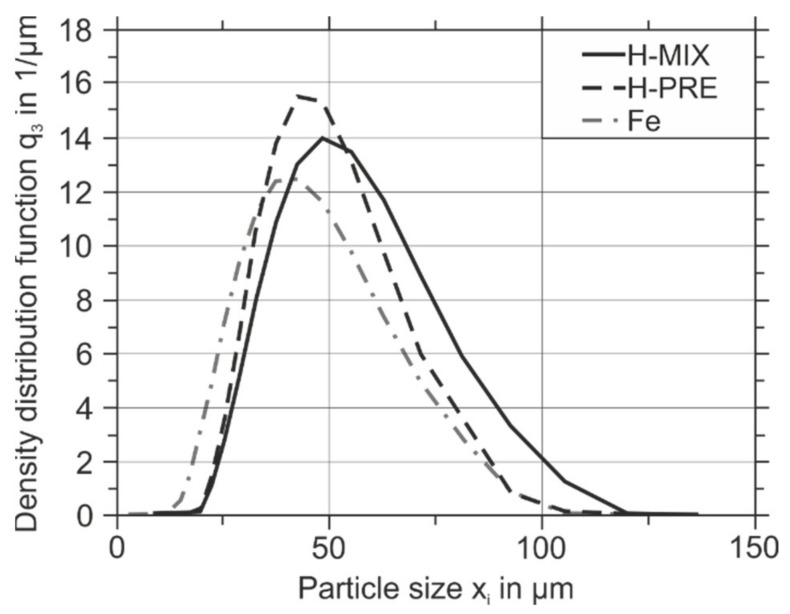
Particle size distributions of H-PRE, H-MIX, and Fe powder.

The particle size distributions (PSD) of H-PRE, H-MIX, and Fe powder are shown in Figure 3. The characteristic particle sizes of H-PRE, H-MIX, and Fe powder are given in Table 3. Fe had the narrowest PSD of the three powders. In contrast, H-MIX had the broadest PSD. The admixed particles were, on average, larger than the Fe particles and therefore the PSD broadened by admixing of the broken particles. Due to the irregular shape of the broken particles, some particles with a size above 63 µm can be recognized in the PSD. Depending on the orientation of those irregular-shaped particles they can pass the sieve. A similar effect can be observed for elongated gas-atomized H-PRE particles.

In summary, the addition of milled particles to gas-atomized pure Fe leads to a reduction in circularity and a broadening of the PSD. Due to the high sphericity of the Fe powder, the average sphericity of H-MIX is only slightly lower compared to H-PRE.

**Figure 4:**
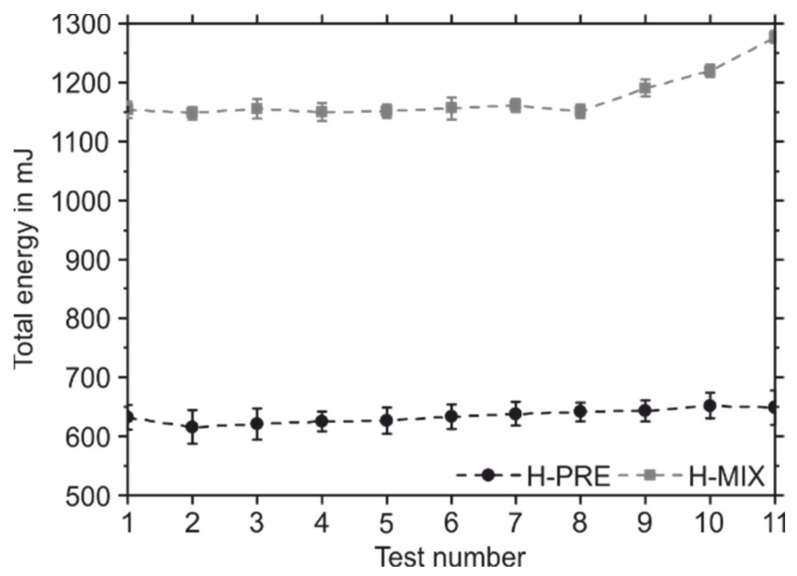
Total energy needed for the movement of the rotating blade into the powder containing vessel of H-PRE and H-MIX.

**Figure 5:**
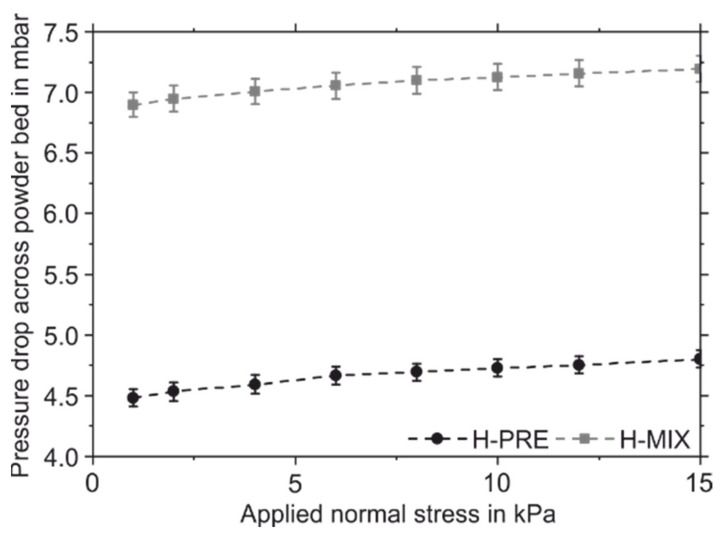
Pressure drop across the powder bed of H-PRE and H-MIX for different applied normal stresses.

Different powder properties were observed by using the FT4 powder rheometer. Figure 4 shows the total energy required to move the rotating blade into the vessel containing the powder sample and its standard error of the mean value over eleven measurements. The first eight measurements were performed with a blade tip speed of 100 mm/s. Subsequently, the blade tip speed was reduced stepwise with a step size of 30 mm/s. Figure 5 shows the pressure drop across the powder sample for different applied normal stresses and its standard error of the mean value. Table 4 contains the values for CBD, BFE, SE, FRI, and PD at a normal stress of 15 kPa and their standard error of the mean values.

The powder-mix shows a lower CBD than H-PRE. This indicates that the gas-atomized H-PRE is packed more efficiently and has less air inclusions, which is favourable for the PBF-LB/M-process [19]. The BFE describes how much energy is required to rotate the blade through the powder sample. A low BFE means that the powder has low resistance to the movement of the blade. The lower BFE of H-PRE compared to H-MIX is an indication of better flow properties and is therefore advantageous for the PBF-LB/M-process [19,34]. While the BFE is measured during the downward movement of the blade, the SE is measured during the upward movement. Here, the powder can move more freely in the axial direction. The SE thus provides information about mechanical interlocking and cohesive forces between powder particles. H-PRE has a lower SE than the H-MIX, which indicates less mechanical interlocking between the particles and lower resistance to flow [19,34]. The FRI describes how the total energy required for the movement of the rotating blade into the powder sample changes when the blade tip speed is reduced. The higher FRI of H-MIX means that when the blade tip speed is reduced, the energy required to move the rotating blade into the sample increases compared to H-PRE. This suggests that H-MIX is more susceptible to changes in coating speed than the gas-atomized powder [34]. At higher blade tip speeds there is more air between the powder particles, which acts as a lubricant. Therefore, the effect of mechanical interlocking is less pronounced at higher blade tip speeds [35]. The higher FRI of H-MIX can be related to pronounced mechanical interlocking of the sharp-edged broken particles compared to the gas-atomized H-PRE.

The permeability of a powder describes its ability to release trapped gas [19,34]. Compared to H-PRE, H-MIX has a higher pressure drop across the powder sample, which corresponds to a lower permeability of H-MIX. This indicates a lower number of channels between the powder particles or smaller channel size and indicates stronger cohesion between the particles of H-MIX compared to H-PRE [34,36]. Low permeability can also result from a high packing density of a powder, but CBD measurements showed that H-MIX was less densely packed than H-PRE [19].

In summary, the flowability tests using the FT4 show that the H-MIX powder has lower CBD, inferior flow properties, a greater dependence of flow properties on blade tip speed, and lower permeability than the gas-atomized reference powder H-PRE. The aspherical particles result in a less efficient packing of the powder. Due to the aspherical shape, there is more mechanical interlocking between the particles, which requires more energy to move the rotating blade through the powder sample. Overall, the powder properties indicate that H-PRE powder is more suitable for the PBF-LB/M-process than H-MIX.

**Figure 6:**
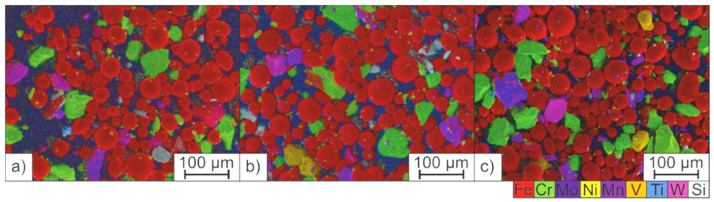
Qualitative EDS mappings of the H-MIX powder bed; (**a**) front, (**b**) centre, and (**c**) rear area of the build platform.

However, the application of dense powder layers could be achieved with both starting powders H-PRE and H-MIX. Specimens of the H-MIX powder bed surface were extracted by using adhesive carbon pads. SEM images superimposed with EDS mappings of these powder samples extracted from the powder bed after the application of 10 layers are shown in Figure 6. The samples were taken from the front, the center and the rear area of the build platform in relation to the direction of the powder application. No differences in the distribution of the individual raw material particles between the three investigated positions on the build platform were observed. However, the particles that were present in smaller amounts than the Cr-rich or Fe particles, such as Ni and FeTi, were found only sporadically inside the powder bed (compare Table 2). A small percentage combined with a big particle size compared to the hatch distance of 60 µm and the laser focus diameter of 30 µm may lead to inhomogeneous local chemical compositions of the PBF-LB/M manufactured samples.

### 3.2. Laser Reflectance Measurement

**Figure 7:**
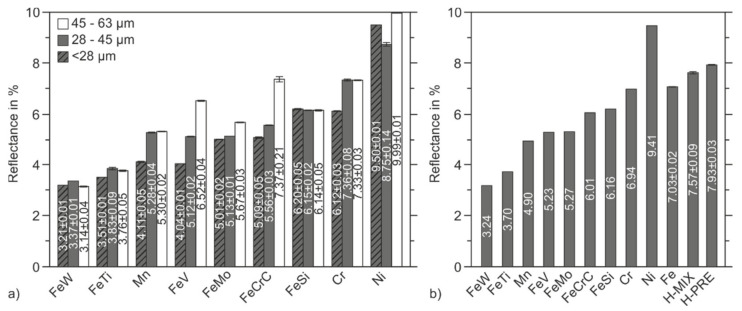
Reflectance of different materials (**a**) elemental powders and (**b**) average reflectance of the three fractions of elemental powders and ferroalloys and reflectance of Fe, H-MIX, and H-PRE in the fractions 20–63 µm.

The laser reflectance of the elemental and ferroalloy powders in three different fractions (<28 µm, 28–45 µm, and 45–63 µm) are shown in Figure 7. Figure 7b shows the average values for the reflectance of the three fractions considered and also contains the values for the Fe powder as well as H-MIX and H-PRE. It has been described in the literature that smaller particles absorb laser radiation more efficiently than larger ones; therefore, particles with a size < 28 µm are expected to reflect less laser radiation than particles of 28–45 µm, which in turn are expected to reflect less laser radiation than particles of 45–63 µm [23,25]. While Mn, FeV, FeMo, and FeCrC showed the behavior described in literature, this behavior was not observed for all investigated materials.

As described in Section 3.1, only a small difference in the particle sizes and morphologies between the fractions 28–45 µm and 45–63 µm can be observed in the SEM images of Mn. This corresponds to the laser reflectance of these two fractions. The 28–45 µm fraction reflected slightly less radiation than the 45–63 µm fraction (5.28% and 5.30%, respectively). FeW and FeTi are the two materials which included a large fine portion in all fractions. Consequently, both materials reflected the lowest amount of laser radiation in all fractions compared to the other materials. For Ni, Cr, FeSi, FeTi, and FeW, smaller particles did not lead to lower reflectance. As can be seen from the example of FeW (Figure 2g–i), finer particles may remain in the larger fractions after dry-screening and thus reduce the reflectance of these fractions. A meaningful relationship between the size of the particles and the reflectance of the fractions cannot be established for sieve-classified powder materials with high amounts of fine portions remaining in the larger fractions.

**Figure 8:**
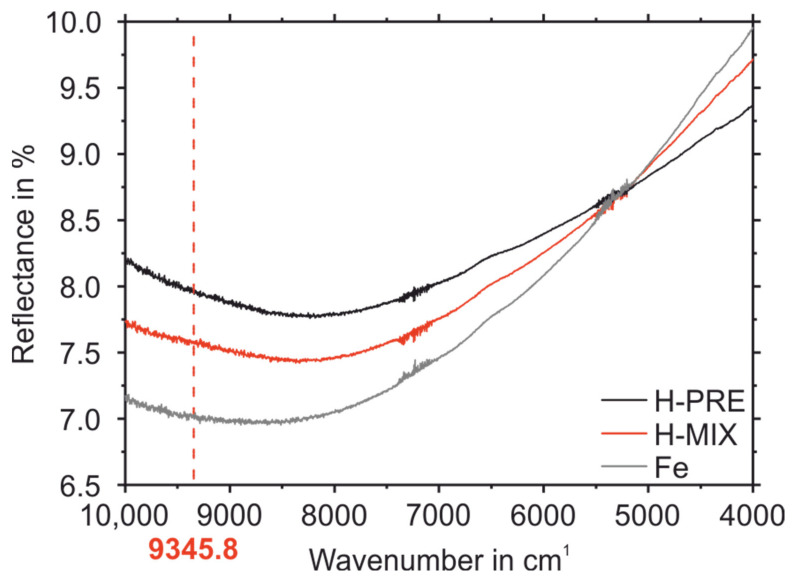
Reflectance spectra of H-PRE, H-MIX, and Fe.

Figure 8 shows the wavelength-dependent reflectance measurements for H-PRE, H-MIX, and Fe (20–63 µm). At a wavenumber of 9345.8 cm^−1^, which corresponds to the constant wavelength of the laser used in the PBF-LB/M-system, H-PRE reflected stronger than H-MIX (compare Figure 7b). The stronger reflectance of H-PRE indicates that H-MIX absorbs laser radiation more efficiently owing to a higher number of beam traps. Thus, to absorb the same amount of energy, a lower applied volume energy density is required. This is associated with a higher scanning speed at constant values for laser power, layer thickness, and hatch distance or a lower laser power at constant values for scanning speed, layer thickness, and hatch distance. Thus, a high laser absorption is desirable for the PBF-LB/M-process from an economic point of view, because energy costs and production time can be reduced this way. Except for Ni, all the elemental and ferroalloy powders studied show lower mean values of reflectance than pure Fe. This leads to the expectation that the reflectance of the mixture H-MIX would be lower than that of Fe. If the reflectance of Fe and the mean values of the reflectance of elemental and ferroalloy powders are added up, weighted by their volumetric proportion in the H-MIX powder (see Table 2), the expected value for the reflectance of H-MIX would be 6.81%. In fact, the measured values of reflectance show that H-MIX (7.57%) reflectance was higher compared to Fe and the calculated value of reflectance of H-MIX. H-MIX consists mainly of pure Fe as the base powder (76.75 vol.%). Pure Fe powder has a lower reflectance than H-MIX, which means that the addition of elemental and ferroalloy powders increased the reflectance compared to pure Fe. This shows that the reflectance of a powder mixture cannot be calculated from the weighted sum of the individual reflectance of the components. The uniformity of a powder bed plays an important role in the reflectance of the powder bed surface [22]. Irregularities in a powder bed can cause fewer reflections between powder particles, which reduces the absorptivity of the powder layer. It is assumed that the addition of broken particles reduced the uniformity of the powder bed surface compared to Fe and resulted in a less dense powder bed surface, thus increasing the reflectance of H-MIX compared to Fe.

### 3.3. PBF-LB/M Densification Behavior of the Tool Steel Powders

**Figure 9:**
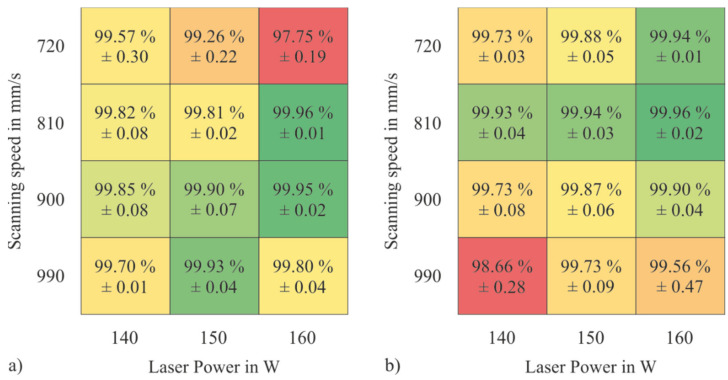
Relative densities of PBF-LB/M-samples manufactured at different process parameters (**a**) H-PRE, (**b**) H-MIX.

**Figure 10:**
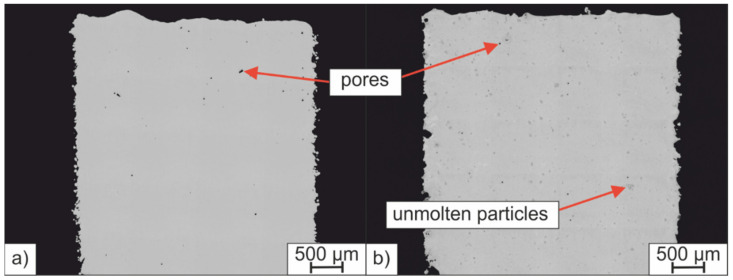
Side-view cross-section of samples manufactured with 160 W laser power and 810 mm/s scanning speed; (**a**) H-PRE, (**b**) H-MIX.

To find suitable process parameters for manufacturing dense sample, a PBF-LB/M parameter study was carried out in which the scanning speed and the nominal laser power were varied between 720 mm/s and 990 mm/s and 140 W and 160 W, respectively. Figure 9 shows the relative sample densities of the PBF-LB/M samples made of H-PRE and H-MIX and their standard deviation. The fields of the tables are highlighted with a color scale. Green indicates a high relative density and red a low relative density. Samples made of both powders showed their highest measured relative densities at 810 mm/s scanning speed and 160 W laser power. Although the flow properties, CBD, and permeability indicate that H-PRE is better suited for the PBF-LB/M-process than H-MIX, both materials achieved a similar maximum relative density of 99.96%. This shows that a lower CBD value, higher values for BFE, SE, and FRI, and a higher PD do not necessarily mean that such a powder leads to lower relative sample densities. The high maximum relative density of the H-MIX PBF-LB/M samples indicates that the quality of the applied powder layers was sufficient. Figure 10 shows the side-view cross-sections of samples produced with a laser power of 160 W and a scanning speed of 810 mm/s. The sample made of H-MIX (Figure 10b) shows unmolten powder particles.

**Figure 11:**
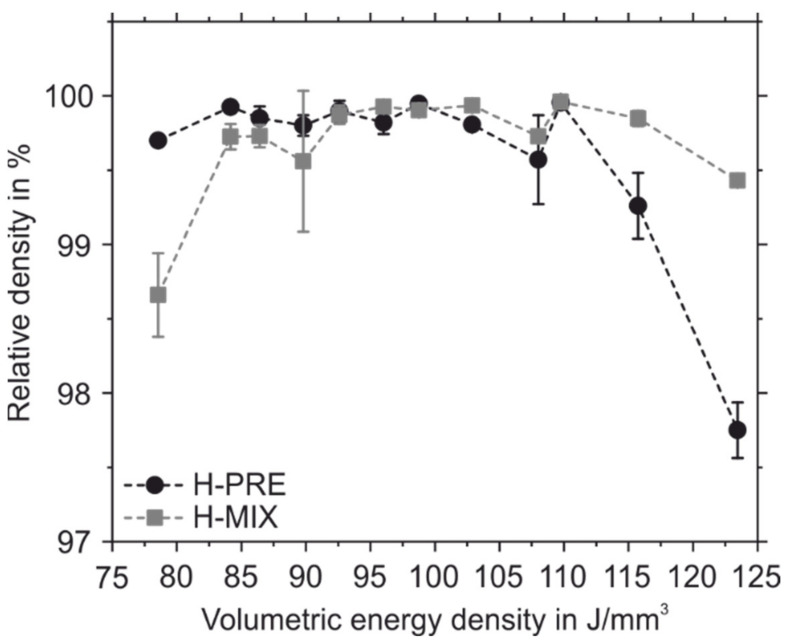
Relative densities of PBF-LB/M samples plotted against the applied volume energy density for H-PRE and H-MIX.

For each applied laser power at lower scanning speeds (720 mm/s and 810 mm/s), samples made of H-MIX showed equal or higher relative densities compared to the samples made of H-PRE. Conversely, for each laser power, samples made of H-PRE achieved higher relative densities at scanning speeds of 900 mm/s and 990 mm/s than samples made of H-MIX. Figure 11 shows the relative densities of the samples made of H-MIX and H-PRE plotted against the applied volumetric energy density. At higher volumetric energy densities, samples made of H-MIX had higher relative density values and at lower applied volume energy densities, samples made of H-PRE were denser. The lower reflectance and thus better absorption of H-MIX at a wavelength of 1070 nm suggests that samples made of H-MIX would show higher relative densities than samples made of H-PRE at faster scanning speeds and lower volumetric energy densities as well, since laser radiation is absorbed more efficiently. A possible explanation for the fact that the H-MIX powder resulted in lower relative sample densities compared to the H-PRE powder at lower applied volumetric energy densities is that the H-MIX powder is composed of different raw materials with different T_LIQ_. Some of these (FeMo, FeW, FeV, Cr, and Fe) possess a higher T_LIQ_ than the H-PRE alloy (compare Table 2 and T_LIQ H-PRE_ = 1458 °C). H-MIX is mainly composed of technically pure Fe powder, which has a T_LIQ_ of 1538 °C. The calculated T_LIQ_ of H-PRE is 80 K lower than that of pure Fe. H-MIX particles must therefore absorb more laser energy to reach the higher T_LIQ_ and be liquefied completely. At lower volumetric energy densities, the amount of absorbed laser radiation is not sufficient to achieve highly dense samples with H-MIX powder. Insufficient amounts of liquid phase are formed to achieve complete densification in the PBF-LB/M process. The need for higher laser energy inputs to produce highly dense samples of H-MIX is not compensated for by its decreased laser reflectance compared to H-PRE (compare Section 3.2).

### 3.4. Solidification and Microstructure of the PBF-LB/M-Fabricated Tool Steel

**Figure 12:**
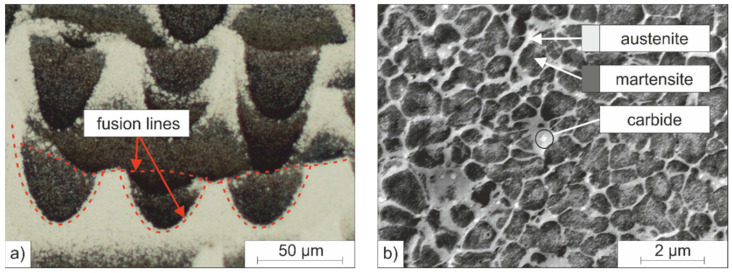
(**a**) Layer-like microstructure of the PBF-LB/M-fabricated samples at lower magnification; (**b**) cellular microstructure of the individual cells formed at a higher magnification, consisting of bcc nuclei surrounded by fcc seams.

The microstructure formation of the investigated tool steel during PBF-LB/M fabrication is described below. The microstructure of the PBF-LB/M samples which were manufactured from the pre-alloyed powder is shown in Figure 12 at two different magnifications and has a hierarchical structure on different size scales. Figure 12a shows a light microscopic image in cross-section in which the melt tracks resembling the alternating scanning pattern are visible due to metallographic etching (the bright and dark areas are etching effects; the melt tracks are marked with hatched lines). Figure 12b shows the cellular substructure at a higher magnification, which is typical for PBF-LB/M-fabricated carbon martensitic hot work tool steels [7]. This substructure is characterized by a high dislocation density, which forms appropriately arranged small-angle grain boundaries, as demonstrated in the work of Geenen using TEM investigations [37].

The individual cells are also hierarchical and consist of a cell nucleus and a cell seam, i.e., the interdendritic region, as shown in Figure 12. Geenen who investigated other carbon martensitic hot work tool steels, reports that the cell nucleus consists of a bcc Fe phase (δ-ferrite, martensite), while the interdentritic region surrounding the cells consists of fcc RA [10]. In accordance with the work of Geenen, Großwendt et al. proved the presence of fcc RA seams surrounding bcc cells for the considered steel after PBF-LB/M-processing in a previous study by EBSD investigations [38].

**Figure 13:**
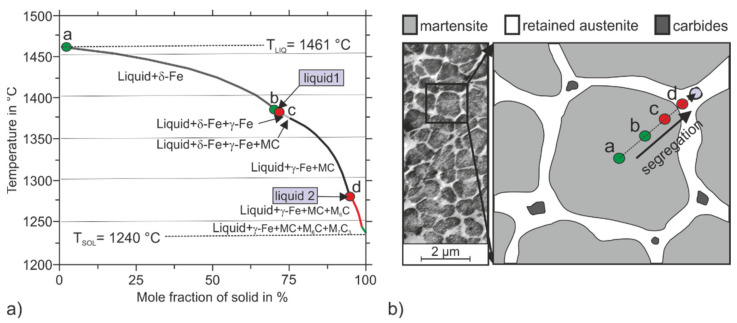
(**a**) Scheil–Gulliver simulation to describe the solidification sequence of the hot work tool steel considered here; points represent calculation locations for determining the local chemical composition. (**b**) Schematic representation of the equiaxial dendritic cellular structure consisting of martensite cells surrounded by RA.

The formation of this cellular or equiaxial dendritic structure of bcc martensite and fcc RA is associated with segregations of the alloying elements on the sub-microscale during the rapid solidification in the PBF-LB/M-process. To illustrate the solidification path and the chemical compositions of the formed phases, a Scheil–Gulliver simulation was carried out. Therein, back diffusions of alloying elements in already-solidified phases were restricted to resemble the rapid cooling rate. It is assumed, that the primary crystallization of δ-ferrite is not suppressed by the rapid cooling rates present in the PBF-LB/M process for the investigated tool steel [7]. Although it has to be mentioned, that Chou et al. suggested a shift to a primary crystallization of fcc austenite for an H13 hot work tool steel based on calculations of austenite and ferrite dendrite tip temperatures at rapid cooling rates [39]. The performed Scheil–Gulliver solidification simulation is shown in Figure 13, wherein the formed phases are plotted against the temperature. Four characteristic points are drawn in the simulations, which mark the beginning (a) and end (b) of ferritic solidification and the beginning (c) and end (d) of the austenitic solidification, where no carbide formation of M_6_C and M_7_C_3_ type takes place. At these characteristic points, the chemical compositions of the ferritic and austenitic phases were calculated and the results are listed in Table 5. The chemical composition was also calculated in the liquid phase to describe the accumulation of alloying elements in the residual melt. The calculated chemical composition in the residual melt for the marked point “liquid 1” in Figure 13 corresponds to the phase transition from δ-ferrite to austenite at 1381 °C. The calculation point “liquid 2” corresponds to the chemical composition in the residual melt at a temperature of 1274 °C, before the eutectic formation of the carbides of type M_6_C and M_7_C_3_ takes place.

According to the Scheil–Gulliver simulation, primary δ-ferrite is formed when the temperature falls below the liquidus temperature of T_LIQ_ = 1461 °C. The primarily formed δ-ferrite possesses a low C content at point a, as shown in Table 5. The other alloying elements, in particular Mo and W, are also lower in the primarily formed δ-ferrite at point a compared to the nominal composition of the steel under consideration. With increasing solidification of δ-ferrite (from point a to point b), the residual melt accumulates preferentially with the elements Cr, Mo, W, and Ni (compare Table 5, point liquid 1). When the temperature falls below 1381 °C, the δ-ferrite, which is primarily formed, is transformed into austenite. Subsequently, only the austenite phase solidifies, which has a higher C content of 0.41 mass% at 1380 °C (see Table 5, point c) compared to the ferrite formed previously. In the further course of solidification, austenite solidifies increasingly and the residual melt is further enriched in the elements Mo, Cr, W, V, Ti, and C. If the temperature falls below 1274 °C, the residual melt has a C content of 1.42 mass% and enriched V- (0.64 mass%) and Ti concentrations (0.44 mass%) which promote the formation of MC carbides (see point liquid 2). Further solidification is characterized by the formation of austenite and V- and Ti-rich MC, until the eutectic concentrations to form Mo- and W-rich M_6_C at 1274 °C and Cr-rich M_7_C_3_ at 1240 °C are reached. Carbides preferably form in the triple points of the cellular structure as marked in Figure 13b which depicts the spatial distribution of the calculated chemical compositions (points a–d) of the phases in a cross-section of the equiaxial dendritic microstructure.

After complete solidification of the melt, the material cools down, considering the respective process parameters (laser power, exposure strategy), the sample size (2D/3D heat dissipation), and the material properties (thermal conductivity, heat capacity). Thereby the previously formed δ-ferrite is transformed into austenite before the local formation of martensite takes place if the material reaches a temperature below the local M_S_ temperatures [7]. The local M_S_ temperatures in relation to the local chemical compositions are approximated using the empirical approach of Andrews (*EQU1*). Accordingly, the formation of the martensite phase starts in the centers of the cell nuclei, as illustrated schematically in Figure 13b. This area solidified first and is rich in Fe but depleted of alloying elements and therefore exhibits the highest local M_S_ temperature of 309 to 349 °C (see points a and b in Table 5). In contrast, the later crystallized intercellular region possesses higher contents of the alloying elements C, Mn, and Ni, which are associated with a strong local reduction in the M_S_ temperature into the range from 81 °C to 220 °C (see points c and d in Table 5). Here it must be taken into account that the martensite formation begins below the M_S_ temperature and is fully completed when the temperature falls below the martensite finish (M_f_) temperature. With these high alloy contents in the intercellular area, the M_f_ temperature is below RT, so that unconverted austenite (RA) remains stable in the microstructure. The amount of RA, depending on the chemical composition of the points a, b c, and d, was roughly approximated with the Koistinen and Marburger approach (Equation (2)) [40].

(2) $$f=exp^{-1\left[1.1\cdot 10^{-2}\cdot \left(M_{S}-T_{U}\right)\right]}$$

The calculated amount of RA is listed in Table 5. The volume fraction of RA increases by decreasing the M_S_ temperature from approximately 3.8–5.6 vol.% (points a and b = cell nucleus) to 17.6–55.0 vol.% (points c and d = intercellular area). The observed cellular structure can be understood via the formation of the RA depending on the local chemical composition (segregations) and the associated M_S_ and M_f_ temperatures. However, martensite transformation not only depends on the chemical driving force but also on local stresses, grain size, and other factors. Additionally, it must also be considered that heat is again inserted into previously solidified and cooled material due to the layer-wise deposition of material, which results in a further austenitizing of areas which were previously transformed into martensite. A heat insertion below the local Ac_1_ or Ac_3_ temperature can cause recovery, recrystallization, the formation of secondary carbides, and partitioning effects [41,42].

As a result of the described solidification sequence and solid-state transformations, a microstructure consisting of martensitic cells surrounded by RA in which carbides are located is formed. The overall RA content was determined using the X-ray diffraction on the PBF-LB/M-produced samples of H-PRE to 21.74 vol.% (see Table 6). This experimental RA volume content is higher than the calculated RA content in Table 5 using the approach of Koistinen and Marburger. The differences can be attributed to the inadequate description of the effect of the elements W, Si, V, and Ti, which are not included in the empirical approach for calculating the M_S_ temperature according to Andrews’ approach, which is used for the calculation of the RA content by Koistinen and Marburger.

**Figure 14:**
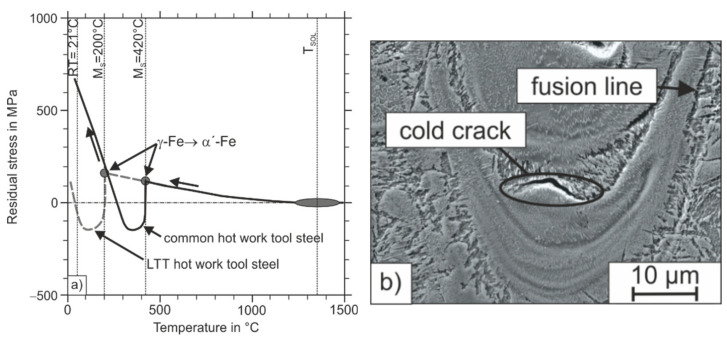
(**a**) Schematic description of the residual stress development in PBF-LB/M-fabrication of carbon martensitic steels; (**b**) microstructure of the sample H-MIX with small cold crack formation.

The low M_S_ temperature and the associated RA content are the reason why this carbon martensitic hot work steel can be processed by PBF-LB/M without cold cracking and strong distortion. An explanation is provided by the schematic drawing in Figure 14, which shows the residual stress development of two carbon martensitic steels (differing in M_S_ temperature) during cooling from the process heat. The solid curve corresponds to a common hot work steel such as X40CrMoV5-1, which exhibits an M_S_ temperature in the range from 250 to 350 °C [7]. With such a steel, residual tensile stresses initially develop as a result of the thermal dilatation during the rapid cooling of the before-formed austenite phase. The formation of thermal residual stresses is more pronounced, the higher the modulus of elasticity, the temperature difference, and the linear thermal expansion coefficient are, and the less time the material has to reduce these thermal tensile stresses, which are formed due to plastic flow during cooling. When the M_S_ temperature is reached, martensite formation begins. Due to the lattice conversion from fcc (packing density 74%) to tetragonally distorted bcc (packing density close to 68%), the material expands, which counteracts the tensile residual stresses previously formed during cooling. This amount of stress released by the formation of martensite represents the so-called transformation-induced plasticity, as occurs in TRIP steels [42]. With further cooling, the martensite volume content increases steadily and thermal residual stresses develop again. Because of the high strength of the martensite, these residual stresses, which have formed again, cannot be reduced by the plastic flow of the martensitic microstructural areas. The stress reduction is therefore limited to the softer RA, which fails by cracking if the stresses are exceed its lower strength. In the LTT concept considered here, the M_S_ temperature is reduced in such a way that the maximum effect of the transformation-induced plasticity can be exploited (compare the hatched line in Figure 14). Because no further thermal residual tensile stresses develop if the M_S_ temperature is set low enough, cold cracking or distortion is avoided. As shown in Table 6, in the hot work tool steel considered here, residual compressive stresses of −142 ± 83 MPa could be measured at RT using the cos-α method, which underlines the justification presented above.

**Figure 15:**
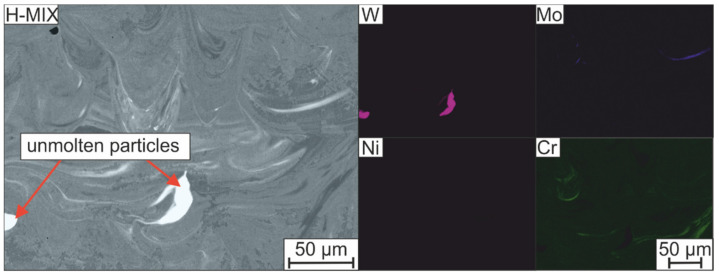
Qualitative studies on the distribution of selected chemical elements in the H-MIX sample using EDS mappings on the left side. Right side shows the area in back scatter electrons (BSE) contrast.

So far, the solidification sequence and the associated microstructure that forms during PBF-LB/M-fabrication of the considered hot work tool steel have been discussed based on the H-PRE samples that were produced from a pre-alloyed gas-atomized feedstock. The microstructure of the H-MIX samples is shown in Figure 14b and Figure 15. Like H-PRE, H-MIX exhibits a hierarchical microstructure consisting of melt tracks on the mesoscale and a cellular microstructure on the microscale. However, in contrast to the microstructure of H-PRE, the micrograph of H-MIX (Figure 15) reveals a bright irregularly shaped phase, which turns out to be W-rich (85 mass% W) by EDS measurements.

Analogous to the element W, EDS mappings in Figure 15 also illustrate the segregation of the other main alloying elements Mo, Ni, and Cr. The strong segregations observed can be attributed to the high-melting ferroalloy particles FeW and FeMo as well as to the pure Cr used for mixing the H-MIX feedstock. These materials possess high liquidus or melting temperatures in the range of 1895–2719 °C (see Table 2). It can be deduced that on one hand, the volumetric energy introduced into the powder bed during the PBF-LB/M-process was sufficient to create a dense microstructure. On the other hand, the applied energy input was inadequate to achieve complete intermixing of the individual alloying elements in the melt pool. Contrary to pure Cr, the usage of pure Ni in the H-MIX feedstock results in less pronounced segregations, which can be linked to the lower melting temperature of Ni of 1455 °C compared to Cr (1907 °C). Therefore, to avoid the observed chemical inhomogeneities caused by insufficiently molten particles, the use of lower melting raw materials can be considered. Moreover, adapted exposure parameters (e.g., higher energy density, multiple exposures) or the use of a laser beam with a larger focus diameter can be considered [43]. Thereby, appropriate melt convection by a sufficient large melt pool for enough time could be achieved [44]. At the same time, finer raw material particles can be used, so that there is a better distribution of the elements in the applied powder bed before exposure. If smaller particles are used, the increased laser absorption of those particle fractions that were investigated in this study could also be utilized to further support the dissolution of higher melting raw materials (compare Section 3.2). Finally, the chemical homogenization could also be increased by a subsequent heat treatment [27]. These measures for increasing the chemical homogeneity of the additively manufactured hot work tool steel will be investigated in future works.

**Figure 16:**
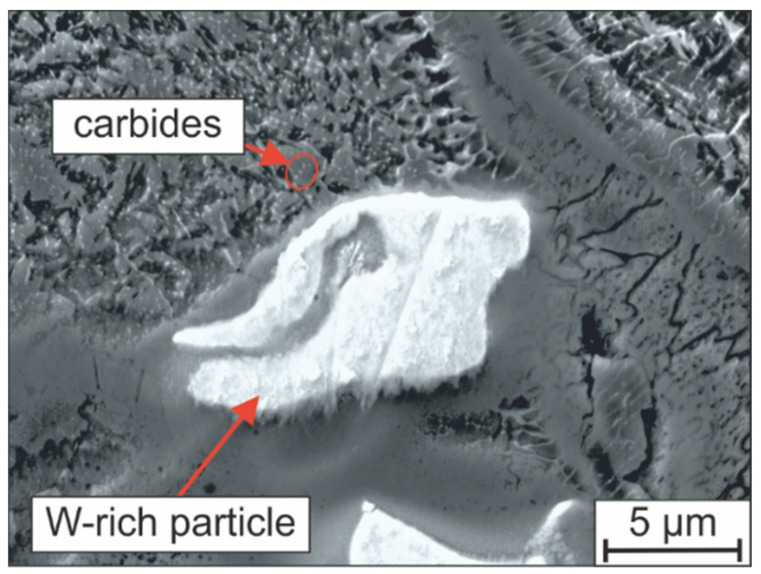
Microstructure of H-MIX in the surroundings of a W-rich particle.

In addition to the undissolved raw material particles, increased numbers of fine carbides can be observed near the unmolten FeW and FeMo particles, as shown in Figure 16. These carbides have a size of a few nm and therefore cannot be analyzed with the analytics installed on the SEM used. In addition to the more inhomogeneous element distribution, a higher RA content of 27 vol.% could be measured in the sample H-MIX compared to the sample H-PRE using X-ray diffraction. According to the previous explanations regarding the stress evolution in LTT steels during the cooling period from the process heat (see Figure 14), this high RA-content is associated with the development of residual compressive stresses because of the martensite formed at lower temperatures and the related transformation-induced plasticity (TRIP) effect. However, because less austenite transforms to martensite, this TRIP effect is less pronounced, which is associated with the formation of lower residual compressive stresses (compare Table 6) [42]. Despite the high RA content, local crack formation can be observed in the microstructure of the H-MIX sample (Figure 14b). The cracks propagate in the RA next to areas where increased martensite formation took place. This locally increased martensite formation is also attributed to the inhomogeneous distribution of the chemical elements associated with the occurrence of incompletely dissolved raw material particles, so that a locally increased chemical driving force for martensite formation due to higher M_S_ temperatures was present. However, the cracks described above are limited to small sample volumes and do not propagate across several melt tracks. The reason for this behavior is the macroscopic compressive stress state of the sample, which counteracts crack growth.

For a possible explanation for the overall higher RA content in sample H-MIX, the unmolten ferroalloy particles and pure elements will be considered again. The elements Cr, Mo, and W are strong carbide formers and form carbides of the types M_23_C_6_, M_7_C_3_, M_2_C, and M_6_C. However, because the hard-phase-forming elements are heavily segregated due to incomplete melting, only partially molten ferroalloy particles can react with the C in the liquid to form carbides. It is assumed that a thin carbide seam is formed around the individual ferroalloy particles. Further carbide formation is suppressed because of the reduced diffusion rate of C in these carbide seams. As a consequence, a stronger enrichment of the residual melt by C of the sample H-MIX during solidification can be assumed. This is accompanied by a higher C-content of the primarily crystallized austenite phase, even if the overall C-content measured in sample H-MIX is lower compared to sample H-PRE (see Table 1). M_S_ temperatures are reduced locally and the amount of RA is increased because of the enrichment of the residual melt by C during solidification (see Equations (1) and (2) and Table 5).To confirm this assumption, the chemical composition was measured selectively in a chemically homogenous metal matrix area in the H-MIX PBF-LB/M sample by means of EDS. The results of such an EDS measurement are listed in Table 7 as representative of a large number of analog measurements. Differences can be found in a reduced W and Mo content in the non-segregated area of the PBF-LB/M sample H-MIX compared to the nominal chemical composition. To fully prove this assumption, the C content in the metal matrix must be measured (e.g., local measurements by wavelength-dispersive X-ray spectroscopy), which will be performed in subsequent studies.

## 4. Conclusions

In this work, the processing of a carbon martensitic hot work tool steel using PBF-LB/M was fundamentally examined. The alloy concept considered is based on the LTT alloy approach. In this alloying concept, the M_S_ temperature and thus the martensite formation is influenced in such a way that samples with low warpage and residual stress can be produced. Samples were produced on the one hand with pre-alloyed gas-atomized powder and on the other hand from a low-cost powder mixture, where the alloying elements are added to a gas-atomized pure iron powder as mechanically broken ferroalloy and elemental particles. This work focused on the reflectance of the laser radiation of the different powders and the microstructure formation in the PBF-LB/M-fabrication. The following key statements can be drawn from the gained knowledge.

- Admixing of gas-atomized spherical Fe powder with aspherical elemental and ferroalloy particles is suitable for the PBF-LB/M-fabrication of highly dense parts.
- PBF-LB/M-samples made of the powder mixture tend to have higher relative densities at higher applied volumetric energy densities than samples made of the gas-atomized pre-alloyed feedstock.
- The powder mixture has a lower CBD, poorer flow properties and lower permeability than the gas-atomized pre-alloyed reference material. Nevertheless, the fact that samples with a high relative density of 99.961% could be manufactured shows that the quality of the powder application during the PBF-LB/M-process is still sufficient.
- Mixing gas-atomized Fe powder with aspherical elemental and ferroalloy powders increases the laser reflectance of the mixture compared to the single reflectance of the components. Still, the mixed powder has a lower reflectance than the gas-atomized, pre-alloyed reference material.
- Feedstocks consisting of very fine particles show the lowest reflectance. Fine portions may remain in larger particle fractions after dry screening and thus reduce the reflectance of those feedstocks.
- Taking the LTT alloy concept into account, hot work tool steels can be achieved which can be processed by PBF-LB/M without preheating. The M_S_ temperature should be below 200 °C so that a RA content higher than 20 vol.% is achieved.
- When using the pre-alloyed gas-atomized powder, crack-free, distortion-free, and low-stress specimens could be manufactured.
- The microstructure possesses a hierarchical built-up. A cellular substructure consisting of martensitic cell nuclei surrounded by austenitic seams can be observed.
- Unmolten particles can be detected in the microstructure which was created from the powder mixture. The energy introduced into the powder bed was not sufficient to achieve a homogeneous element distribution by sufficient melt convection before solidification. The chemical inhomogeneities can counteract the LTT alloying concept and promote cold cracking.

In future work, it shall be investigated how a more homogeneous microstructure can be achieved in PBF-LB/M processing of the tool steel powder mixture. Different exposure strategies (e.g., double exposure), varied mixtures, decreased particles sizes, and post process heat treatments will be considered.

## Figures and Tables

**Table 1:** Chemical composition in mass% of the used hot work tool steel.

Elements	C	Cr	Si	Mn	Ni	Mo	Ti	V	W	Fe
Nominal composition	0.36	10.00	0.70	0.60	1.70	3.00	0.20	0.30	2.00	bal.
H-PRE	0.37 ± 0.01	10.41 ± 0.07	0.75 ± 0.01	0.67 ± 0.01	1.89 ± 0.01	2.97 ± 0.08	0.13 ± 0.00	0.37 ± 0.01	2.89 ± 0.07	bal.
H-MIX	0.29 ± 0.01	8.92 ± 0.06	0.56 ± 0.02	0.50 ± 0.01	1.86 ± 0.07	2.34 ± 0.03	0.09 ± 0.00	0.27 ± 0.02	2.14 ± 0.33	bal.

**Table 2:** Chemical composition of the used ferroalloy particles with a size of 20 to 63 µm in mass%, calculated melting temperature T_m_, and solidus temperature T_sol_ in °C, and the percentage of used ferroalloys and pure elements.

Powder	Circularity	Fe	Cr	Mo	W	Ni	V	Mn	C	Al	Si	Ti	Calculated T_m_ or T_Liq_ in °C	Content in Mass%	Content in vol.%
FeCrC	0.58	28.1	61.2	-	-	-	-	0.3	7.5	-	2.9	-	1563	4.35	4.91
FeMo	0.55	20.3	-	69.7	-	-	-	-	-	-	-	-	1895	4.36	3.87
FeW	0.59	19.0	-	-	80.5	-	-	-	-	-	0.5	-	2719	2.62	1.95
FeV	0.51	17.2	-	-	-	-	80.5	-	0.2	1.4	0.7	-	1447	0.38	0.47
FeTi	0.61	29.1	-	-	-	-	-	-	-	-	-	70.9	1117	0.28	0.40
FeSi	0.51	24.3	-	-	-	-	-	-	-	0.1	75.5	0.1	1191	0.85	1.80
Cr	0.54	-	99.9	-	-	-	-	-	-	-	-	-	1907	7.13	7.77
Ni	0.64	-	-	-	-	99.9	-	-	-	-	-	-	1455	1.69	1.47
Mn	0.55	-	-	-	-	-	-	99.9	-	-	-	-	1414	0.58	0.61
Fe	0.85	99.9	-	-	-	-	-	-	-	-	-	-	1538	77.75	76.75

**Table 3:** Characteristic particle sizes of H-PRE, H-MIX, and Fe powder in µm.

	H-PRE	H-MIX	Pure Fe
d (0.1)	31.60	29.10	25.21
d (0.5)	47.57	46.05	42.41
d (0.9)	71.28	72.33	69.49

**Table 4:** Flowability characteristics of H-PRE and H-MIX powders.

	H-PRE	H-MIX
CBD in g/mL	4.67 ± 0.00	4.43 ± 0.02
BFE in mJ	638.00 ± 19.98	1161.33 ± 11.14
SE in mJ/g	2.32 ± 0.04	3.61 ± 0.03
FRI	1.01 ± 0.02	1.11 ± 0.01
PD @ 15 kPa in mbar	4.80 ± 0.07	7.19 ± 0.11

**Table 5:** Chemical composition in mass% at characteristic solidification intervals, marked in Figure 13.

	T in °C	C	Cr	Mo	W	Ti	V	Si	Mn	Ni	M_s_ in °C	RA in vol.%
a	1461	0.04	9.64	2.45	1.53	0.02	0.18	0.58	0.42	1.42	349.16	3.80
b	1381	0.09	9.77	2.77	1.85	0.03	0.25	0.82	0.61	1.91	309.59	5.60
liquid 1	1381	1.03	10.77	4.13	2.97	0.26	0.47	0.79	0.89	2.26	-	-
c	1380	0.31	9.14	2.07	1.34	0.06	0.19	0.74	0.69	2.32	219.71	13.7
d	1274	0.54	10.72	3.53	2.46	0.04	0.27	1.04	0.96	2.51	80.78	55.0
liquid 2	1274	1.42	11.81	5.50	4.05	0.44	0.64	0.79	0.97	2.14	-	-
nominal	-	0.36	10.00	3.00	2.00	0.20	0.30	0.80	0.60	1.80	193.12	17.90

**Table 6:** Experimentally measured residual stresses inside bcc martensite and retained austenite content for H-PRE and H-MIX.

	RA in vol.%	Compressive Residual Stress in MPa
H-PRE	21.74 ± 1.15	−142.00 ± 82.52
H-MIX	27.24 ± 2.00	−113.33 ± 57.38

**Table 7:** EDS point analysis to measure the local chemical composition in the H-MIX sample in an unmolten FeW particle and in the “homogeneous” metal matrix.

	Cr	Si	Mn	Ni	Mo	Ti	V	W	Fe
Unmolten FeW particles	0.60 ± 0.20	0.00 ± 0.00	0.17 ± 0.09	0.99 ± 0.33	0.00 ± 0.00	0.14 ± 0.14	0.07 ± 0.07	85.30 ± 0.74	7.84 ± 0.73
Matrix	11.4 ± 0.36	0.98 ± 0.10	0.48 ± 0.11	1.97 ± 0.22	1.94 ± 0.39	0.03 ± 0.04	0.42 ± 0.05	0.61 ± 0.05	80.48 ± 0.19
Nominal	10.00	0.70	0.60	1.80	3.00	0.15	0.30	2.00	Bal.

## Data Availability

The data presented in this study are available on request from the corresponding author.
